# Lead isotopic evidence for an old and rapid lunar magma ocean

**DOI:** 10.1126/sciadv.adu5111

**Published:** 2025-08-06

**Authors:** Ya-Wen Zhang, Si-Zhang Sheng, Shui-Jiong Wang, Qiu-Li Li, Jun-Xiang Hua, Shijie Li, Xian-Hua Li

**Affiliations:** ^1^ State Key Laboratory of Geological Processes and Mineral Resources, China University of Geosciences (Beijing), Beijing 100083, China.; ^2^ Frontiers Science Center for Deep-time Digital Earth, China University of Geosciences (Beijing), Beijing 100083, China.; ^3^ State Key Laboratory of Lithospheric and Environmental Coevolution, Institute of Geology and Geophysics, Chinese Academy of Sciences, Beijing 100029, China.; ^4^ Center for Lunar and Planetary Sciences, Institute of Geochemistry, Chinese Academy of Sciences, Guiyang, China.; ^5^ Center for Excellence in Comparative Planetology, Chinese Academy of Sciences, Hefei, China.

## Abstract

The Moon’s early history remains enigmatic, with intensive debate over the timing and duration of the lunar magma ocean (LMO) and its consequences on the early thermal and chemical evolution of the Moon. Here, we report a lead-lead isochron age of 4406.1 ± 3.2 million years ago (Ma) with an extraordinarily high initial lead (Pb) composition (HIP) for a lunar meteorite Northwest Africa 14729. The HIP at ~4406 Ma implies Pb isotopic evolution from an early-formed, high-μ (^238^U/^204^Pb > 5000) reservoir that is genetically linked to the last remaining liquid of the LMO called urKREEP. This requires that lunar primordial differentiation was completed within approximately 150 million years (Myr) after the Solar System’s formation.

## Teaser

Lead isotopes unveil an old and short-lived magma ocean on the Moon.

## INTRODUCTION

A giant impact on the proto-Earth led to the formation of the Moon with a magma ocean (*1*). The classic lunar magma ocean (LMO) differentiation model predicts a sequential formation of a series of cumulates that physically separated (*2*–*4*). After 99% of the LMO solidified, the last remaining liquids evolved to form a subcrustal layer strongly enriched in incompatible elements including potassium (K), rare-earth elements (REE), and phosphorus (P), known as urKREEP (*5*). Despite extensive research over decades, a fundamental question remains surrounding the timing and duration of the primordial lunar differentiation, leading to debate over whether it was an old [~4500 million years ago (Ma)] or young LMO (~4350 Ma), and whether the LMO was long-lived [~150–200 million years (Myr)] or short-lived (~ <30 Myr) (*4*, *6*–*11*). Each of these extremes would have led to a very different outcome during the early thermal and chemical evolution of the Moon.

The formation of a high-μ (^238^U/^204^Pb) urKREEP reservoir is considered a diagnostic indicator of the termination of the LMO (*5*, *12*). However, accurately dating the time when the urKREEP reservoir was isolated from the LMO has proven to be challenging (*11*, *13*–*16*). Chronologic investigations on a variety of KREEP-bearing lunar lithologies, using multiple isotopic systems, have yielded isochron or model ages spanning over 200 Myr. A significant portion of these ages cluster between 4340 and 4380 Ma, which has been interpreted as evidence for a late Moon formation and rapid crystallization of the LMO around 4350 Ma (*7*, *17*, *18*). Nevertheless, this inference of a “young” LMO differentiation event conflicts with the older U-Pb and Hf model ages determined for lunar zircons (*6*, *10*, *11*, *16*, *19*–*23*). Zircon saturation experiments show that only KREEP-rich magma on the Moon has sufficient zirconium to produce zircon (*10*, *24*). Therefore, the oldest age of Zr phases sets a younger limit for the time of urKREEP crystallization, pointing toward an early Moon formation within 150 Myr after the Solar System’s formation (*6*, *9*, *11*). Nimmo *et al.* (*9*) proposed that the frequently observed approximately 4350 Ma ages among lunar rocks and zircons are more likely related to a tidally driven remelting event of the Moon rather than the original LMO crystallization. Given this, the mystery of the onset time of urKREEP separation from the LMO remains, as the proposed remelting event would have reset most chronometers and muddles the existing data.

Here, we present a secondary-ion mass spectrometry (SIMS) Pb isotopic investigation on the troctolite clasts in Northwest Africa (NWA) 14729 (see Materials and Methods). The meteorite NWA 14729 is a lunar troctolite melt breccia containing a 3 cm by 9 cm troctolite clast (fig. S1). A detailed description is given in Materials and Methods. In brief, the troctolitic clast, characterized by a cumulate structure, is surrounded or crosscut by fine-grained matrix and shock melts (figs. S1 and S2). NWA 14729 consists of 67.8 to 73.0 vol % plagioclase (with an anorthite content of An_93.5–98.2_), 17.9 to 18.4 vol % olivine (with a forsterite content of Fo_84.0–88.7_), 8.6 to 14.3 vol % pyroxene (exhibiting a Mg# value of 68.5 to 91.4), along with trace amounts of spinel, troilite, and metal. The Fe/Mn ratios of olivine and pyroxene vary from 74.5 to 105 and 33.2 to 68.1, respectively, confirming the lunar origin (fig. S3). The abundances of transition metals in olivine (e.g., Ni, Co, and Cr) fall within the range typical of olivine in lunar troctolites (*25*) (fig. S4). Various Zr phases, including loveringite, baddeleyite, and zircon, are also present and coexist with the silicate minerals. Chemical compositions of major and accessory minerals are reported in table S1.

## RESULTS

**Fig. 1.**
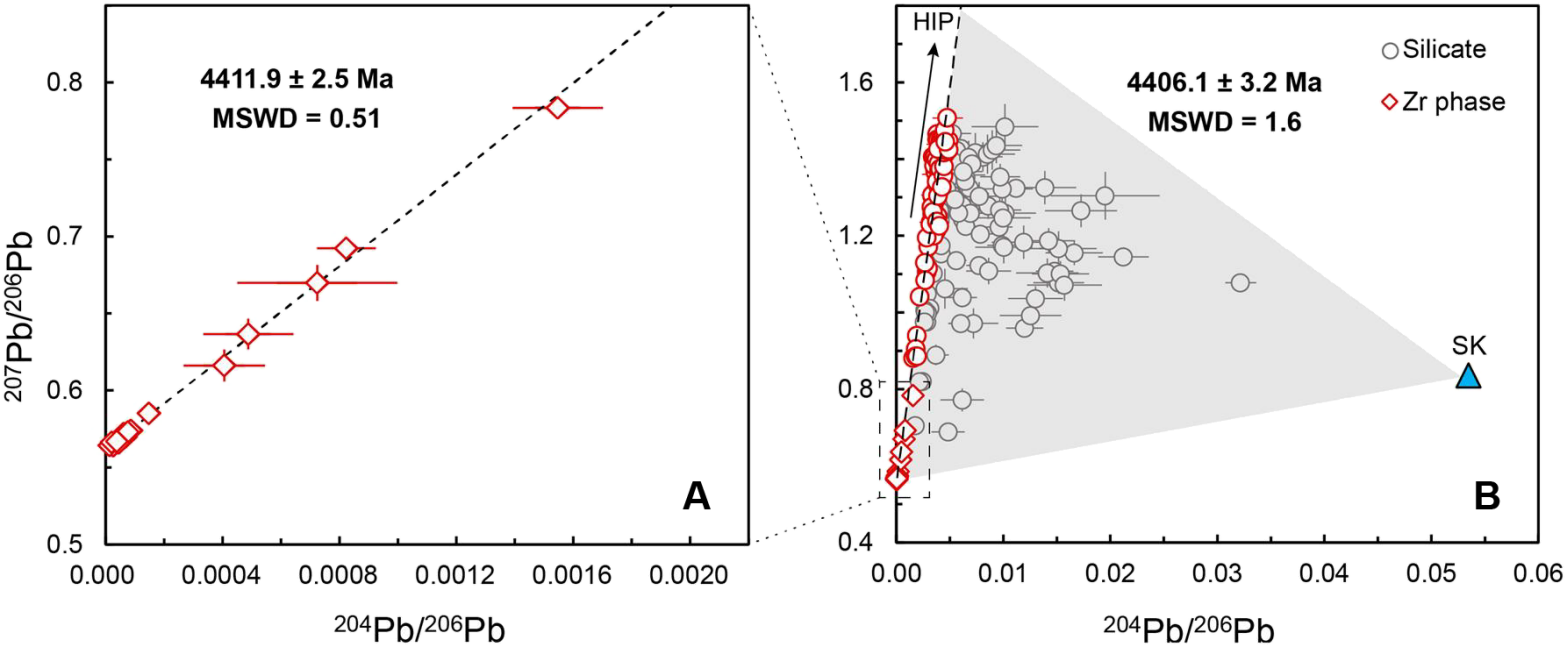
Pb-Pb isochron for NWA 14729. (**A**) ^207^Pb/^206^Pb versus ^204^Pb/^206^Pb plot of the Zr phases. (**B**) ^207^Pb/^206^Pb versus ^204^Pb/^206^Pb plot of all Zr phases and silicates. Filtered data of the silicates to define the isochron are shown in red, whereas others are shown in gray. The modern terrestrial Pb composition (SK) is shown in blue triangle (*26*). Error bars represent 2σ. HIP, high initial Pb composition. Data are presented in table S2.

Twenty-one analyses on 12 Zr phases yield a well-defined Pb-Pb isochron age of 4411.9 ± 2.5 Ma [mean square weighted deviation (MSWD = 0.51)] (Fig. 1A; fig. S5 for morphology of Zr phases). The Pb isotopic compositions determined for the silicate mineral phases, for example, plagioclase, pyroxene, and olivine, mostly fall along this isochron, whereas some of the data show terrestrial contamination as manifested by falling to the right of the isochron and toward the composition of modern terrestrial Pb (*26*) (Fig. 1B). Filtering out potentially contaminated Pb data to identify a leftmost line of the triangle region yields an isochron age of 4406.1 ± 3.2 Ma (MSWD = 1.6; Fig. 1B).

The isochron points toward an extraordinarily high initial Pb composition (HIP; Fig. 1B). The least radiogenic composition measured in the plagioclase of this sample (e.g., spot 7783-11; table S2) may record the composition most approaching to the HIP. However, this value still underestimates the ^207^Pb/^206^Pb and ^204^Pb/^206^Pb ratios of the true HIP. By finding the intersection at ~4406 Ma between different model paleosiochrons and the sample isochron, we constrain the HIP value of ^204^Pb/^206^Pb ≥ 0.00578 and ^207^Pb/^206^Pb ≥ 1.74670 (fig. S6; Materials and Methods for modeling details).

## DISCUSSION

The isochron provides direct evidence for the “old” LMO differentiation occurring no later than ~4406 Ma. The HIP of the isochron is highly radiogenic, indicating that Pb isotopic evolution in an urKREEP reservoir with an extremely high μ value.

**Fig. 2.**
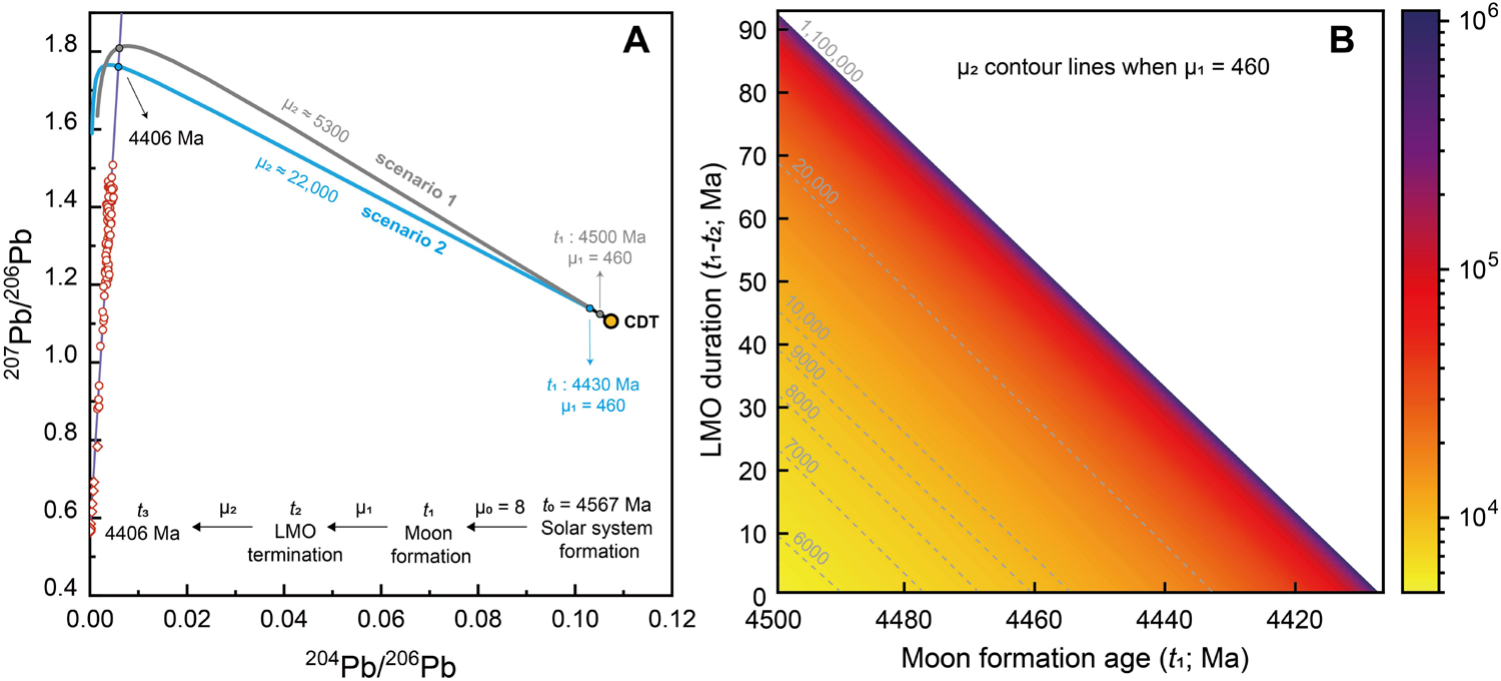
Three-stage Pb isotopic growth model. (**A**) ^207^Pb/^206^Pb versus ^204^Pb/^206^Pb plot, with the insert showing the stages of Pb isotopic evolution. (**B**) Monte Carlo–derived modeled μ_2_ values as a function of the Moon formation time (*t*_1_) and the LMO duration (*t*_1_-*t*_2_). Colored lines represent contour lines of μ_2_ values at μ_1_ = 460 (*32*).

We conducted a three-stage Pb evolution model, as illustrated in the Pb-Pb plot (Fig. 2A; Materials and Methods for modeling details). The first stage starts from the primitive starting composition of Canyon Diablo Troilite (CDT) at ~4567 Ma (*t*_0_) (*27*, *28*) and extends to the time of Moon formation (*t*_1_) with a μ value of 8 (μ_0_) (*29*, *30*). The second stage represents the duration of the LMO, starting from *t*_1_ to the termination of the LMO at *t*_2_, assumed to be the onset of urKREEP reservoir separation. The μ value of the bulk Moon (μ_1_) at this stage is not well constrained but has been suggested to be lower than 1000 [e.g., (*15**,* *31**,* *32*)]. For example, Snape *et al.* (*15*) analyzed the Pb isotopic composition of lunar basalts and estimated the μ_1_ to be ~460 based on Pb isotopic evolution model. The third stage begins with the formation of the urKREEP reservoir (μ_2_) and evolves until 4406 Ma.

Constraining the timing of the separation of the urKREEP reservoir from the LMO depends on the μ_2_ value, which is however even less constrained. We apply a Monte Carlo approach, whereby μ_1_ (8 to 1000), μ_2_ (>μ_1_), *t*_1_, and *t*_2_ are varied randomly, to explore the relationship of μ_2_ with the Moon formation time (*t*_1_) and LMO duration (*t*_1_-*t*_2_). The simulation result is insensitive to the parameter of μ_1_ (fig. S7). At a constant μ_2_ value, the *t*_1_ and (*t*_1_-*t*_2_) are negatively correlated (Fig. 2B), suggesting that the later the Moon formed, the shorter the LMO duration would be. We envision two end-member scenarios for the lunar Pb isotopic evolution. If the Moon formed as early as 4500 Ma (*33*), then the intersection of the isochron with the Pb isotopic modeling curve at 4406 Ma would require a μ_2_ value of at least 5300. At this value, the LMO duration would be extremely short. Increasing the μ_2_ value would still produce a LMO duration less than 94 Myr (Fig. 2B). If the Moon formed slightly later, e.g., at ~4430 Ma (*10*), the Pb isotopic evolution model requires that the urKREEP source has a significantly higher μ value (>22,000) to evolve rapidly to have a HIP at ~4406 Ma, and the LMO duration would be less than 24 Myr regardless of the μ_2_ value (Fig. 2B). Many previous estimates based on Pb isotopic system in lunar basalts suggest that the urKREEP reservoir has a μ value in the range of several thousands (*15*, *31*, *32*, *34*), but extremely high μ values for the urKREEP are not impossible. If the latter is true, a hidden efficient sink of Pb, probably sulfide, must have segregated Pb from the liquid during LMO differentiation, resulting in the extremely high μ value of the urKREEP (*35*). Or, alternatively, the bulk silicate Moon started with a μ_1_ value substantially higher than traditionally thought, as a result of a combined effect of core formation and evaporative loss of Pb during the formation of the Moon (*31*, *36*, *37*). All these deserve further investigation. Nevertheless, the HIP signature at ~4406 Ma strongly suggests that the onset of urKREEP separation, which is equivalent to the termination of the LMO, occurred within the first 150 Myr after Solar System formation.

It has long been recognized that HIP, contributed by the evolved urKREEP component, is widely present in lunar highland rock lithologies (*12*, *15*, *38*–*40*). While the accurate initial Pb value of the urKREEP at a given age is poorly constrained, the HIP determined in this study can establish a lowest limit (Fig. 1B and fig. S6; Materials and Methods for modeling details). Zircon saturation experiments suggest that the growth of Zr phases is closely associated with melts enriched in urKREEP component (*24*). A recent Lu-Hf isotopic study also demonstrated that zircons from the Apollo missions were all derived from a single urKREEP reservoir that was isolated from the bulk silicate Moon (*10*). Therefore, we use the newly determined HIP as an approximation of the initial Pb compositions of lunar zircons and re-correct the ^207^Pb/^206^Pb ratios obtained from previous reported old zircons (>4350 Ma, which were mostly corrected by using modern terrestrial Pb composition) (*11*, *13*, *16*, *19*–*22*, *41*). The HIP-corrected ^207^Pb/^206^Pb ages span continuously in between 4350 and 4406 Ma (fig. S8 and table S3). We suggest that the 4350 to 4406 Ma may represent an important thermal-magmatic period after the LMO crystallization, having induced massive thermal and chemical exchange between the lunar crust and mantle, reset the Moon’s geological clock, and likely had a profound influence on the subsequent lunar evolution.

One possible trigger for the 4350 to 4406 Ma great melting episode may be ancient basin-forming impacts. The large impact would have generated an extensive melt sheet that incorporated the urKREEP component with a HIP signature and other rock lithologies (e.g., Mg-suite rocks and ferroan anorthosites) having variable initial Pb compositions, resetting the U-Pb system. Alternatively, the age may record the time of the first Mg-suite magmatism on the Moon. This inference is supported by the well-defined isochron constructed by the Zr phases and troctolitic minerals (Fig. 1B). The ascending Mg-suite parent magmas incorporated an early formed, evolved high-μ urKREEP component, and inherited the HIP signature. The heat source for this extensive mantle-crust interaction has been proposed to be related to the tidal heating during the Moon’s passage through the Laplace plane transition (*9*). The prolonged nature of the Laplace plane transition (*42*) could have triggered prodigious mantle melting and volcanism several times over a period of tens of millions of years (*9*) and may have been responsible for widespread magmatism in between 4350 and 4406 Ma.

In conclusion, this study shows that the LMO differentiation completed within the first 150 Myr after Solar System’s birth. Our Pb isotopic evolution model further demonstrates that the duration of the LMO was relatively short (<94 Myr) depending on the Moon formation time and the nature of U and Pb fractionation during the LMO differentiation. If the LMO differentiation resulted in an urKREEP reservoir with μ values of several thousand, which is likely the prevailing view, we infer that the LMO started at least 4460 Ma with a very short LMO duration of <50 Myr. The younger ages of 4350 to 4406 Ma recorded by lunar zircons might reflect a great melting episode after the LMO crystallization on the Moon.

## MATERIALS AND METHODS

### Sample description

The meteorite NWA 14729 is a lunar polymict anorthositic troctolite melt breccia, purchased in Morocco in 2021. The largest clast (3 cm by 9 cm) from NWA 14729 is characterized by high magnesium content, as revealed by micro–x-ray fluorescence spectroscopy (micro-XRF) (fig. S1A). Two rock chips were split from this high Mg clast, mounted in epoxy, and polished to make thin sections. Mineralogical information and major elemental distribution maps were obtained by using Tescan integrated mineral analysis (TIMA). The high Mg clast is either surrounded by or intersected with fine-grained matrix mainly composed of quenched silicate glass, along with recrystallized melt pockets and fine-grained silicates (<20 μm). Mineral phases were identified and delineated in different colors based on major elemental distributions (see fig. S1, B and C). The colored elemental maps were then imported into the ImageJ program. By applying the Color Threshold and Analyze Particles functions, the pixel counts within the defined areas were then used to calculate the final modal abundances. The protolith of the high-Mg clast is composed of 67.8 to 73.0 vol % plagioclase, 17.9 to 18.4 vol % olivine, and 8.6 to 14.3 vol % pyroxenes located in the field of anorthositic troctolite (*43*) (fig. S1D). Despite being affected by impact processes, the representative cumulate texture of the anorthositic troctolite remains discernible (fig. S2).

Mineral fragments, including plagioclase, pyroxenes, olivine (0.05 to 2 mm), trace amount of spinel (~0.05 mm), and Zr-bearing minerals (zircon, baddeleyite, and loveringite, 4 to 25 μm), are mixed with a composition approximating that of the aforementioned mineral assemblage. The Fe/Mn ratios of olivine and pyroxenes confirm its lunar origin (fig. S3). Olivine grains are euhedral to subhedral and vary in size from a few micrometers to several hundred micrometers. Olivine grains exhibit a tight compositional range (Fo_84.0–88.7_, *n* = 63; fig. S4A). All olivine fragments have Ni, Co, and Cr contents consistent with olivine in lunar troctolites (*25*) (fig. S4, B and C). Both low-Ca pyroxene and high-Ca pyroxene were observed. Low-Ca pyroxene fragments have a compositional range of Wo_0.8–18.6_En_57.9–88.5_Fs_10.1–30.7_ (*n* = 46), while high-Ca pyroxene fragments range from Wo_27.6–42.8_En_49.6–64.6_Fs_5.0–12.7_ (*n* = 22) (fig. S4D). The Mg# values of pyroxene vary from 68.5 to 91.4. Plagioclases have a tight compositional range of An_93.5–98.2_ (*n* = 48). The Mg# values of the spinel grains vary widely, ranging from 61.8 to 87.1, while the Cr# values form a tight cluster between 3.0 and 5.7 (*n* = 34), which corresponds to the composition of spinel in pink spinel troctolite (*44*) (fig. S4E). Zr-bearing minerals including loveringite (*45*) [with ZrO_2_ content varying from 3.12 to 6.78 weight % (wt %)], zircon and baddeleyite are closely associated with troctolitic clasts or mineral fragments. Some loveringite grains occur as discrete grains, and some form intergrowths with armalcolite (fig. S5). With limited or little intrusion of fine-grained matrix, certain regions retain characteristics representative of the protolith. The compositions of silicates and spinel in A6471 and A6472 indicate that the clast was originated from the lunar highlands Mg suite (fig. S4, A to E). Therefore, the associated Zr-bearing minerals should be considered primary phases within the anorthositic troctolite studied here.

Quenched glasses have Mg# values ranging from 63.9 to 88.1 and Al_2_O_3_ content from 15.4 to 32.7 wt %. Most of the quenched glasses have compositions aligned with Apollo HMS and feldspathic meteorites (fig. S4F), suggesting that the impact may have excavated the deep crustal section and mixed different lithologies including Mg-suite intrusions and ferroan anorthosites. In general, the petrology and geochemistry suggest that the protolith of the high Mg clast in NWA 14729 is an anorthositic troctolite.

### Micro–x-ray fluorescence spectroscopy (micro-XRF)

Micro-XRF experiments were performed on the Bruker M4 TORNADO PLUS μXRF spectrometer at the Institute of Geology and Geophysics, Chinese Academy of Sciences (IGGCAS). In this study, the scanning μXRF element mapping experiment was performed with an x-ray tube energy of 50 kV and a current of 600 μA, with 40 ms per pixel spectrum acquisition time and a pixel step size of 9 μm (*46*). Data analyses including obtaining elemental maps on all objects and XRF spectrum from each object or region of interest were undertaken with the characterization software provided by Bruker Micro Analytics.

### Tescan integrated mineral analysis (TIMA)

Major elemental mappings of thin sections were determined by TIMA at the Key Laboratory of Orogenic Belts and Crustal Evolution, School of Earth and Space Sciences, Peking University, China. The TIMA system comprises of a Tescan Mira Schottky field-emission scanning electron microscope with four silicon-drift energy-dispersive (EDS) detectors arranged at 90° intervals around the chamber. The measurements were performed in the high-resolution liberation analysis mode, and the backscattered electron image was obtained to identify individual particles and boundaries between distinct preliminary phases. A rectangular mesh of measurements on each distinct phase was obtained with x-ray spectra. TIMA was performed at 25 kV using a spot size of ~1 μm, a working distance of 15 mm, and a field size set at 1500 μm.

### Electron microprobe analysis (EPMA)

The mineral major-element compositions were determined using EPMA1720 electron microprobe at the EPMA Lab, China University of Geosciences, Beijing (CUGB), and JEOL JXA8100 electron probe at IGGCAS, respectively. The samples were analyzed at an acceleration voltage of 15 kV, a beam current of 10 nA, and a focused beam width of 1 to 2 μm at CUGB, and an accelerating voltage of 15 kV, a probe current of 20 nA, and a focused beam width of 1 to 2 μm at IGGCAS. The peak counting time was 10 to 30 s for each element and the background time was 10 s. Natural minerals and synthetic materials were used as standards as follows: rhodonite (Si and Mn), apatite (Ca and P), rutile (Ti), FeS_2_ (Fe), albite (Na), sanidine (K), MgO (Mg), V (V), chromite (Cr), garnet (Y), zirconia (Zr), monazite (La and Ce), and ZnS (Zn). All data were corrected for atomic number (Z), x-ray absorption (A), and fluorescence (F) effects. The detection limits for most elements were 0.01 to 0.06 wt %. Data are reported in table S1.

### SIMS Pb isotopic analysis

The Pb isotopic compositions were determined on a CAMECA IMS 1280HR secondary ion mass spectrometry (SIMS) at the IGGCAS. The mounts containing the candidate minerals were cleaned with a fine (0.25 μm) diamond paste and ethanol and then a roughly 20-nm carbon coating was added.

The first session focused on the Pb isotope measurement of Zr phases. A Gaussian illumination mode was used to focus a primary beam of ^16^O^−^ to a size of ~3 μm, with an accelerated potential of −13 kV. The beam size can be kept unchanged for a long usage time and intensities were around 250 to 200 pA. The primary beam setting is described in detail in (*47*). The multicollector mode with four electron multipliers was used to measure ^204^Pb^+^ (L2), ^206^Pb^+^ (C), ^207^Pb^+^ (H1), and ^96^Zr_2_^16^O_2_^+^ (H2). The methodology is similar to that outlined in (*48*). Exit slit 3 was used, with a mass resolving power (MRP) of 8000 (50% peak height). Before analysis, a primary beam of ^16^O^−^ with an intensity of 10 nA was used for 120 s of presputtering. The ion images with ^96^Zr_2_^16^O_2_^+^ and Pb isotopes on a 25 μm by 25 μm area were used to precisely locate the target minerals. The signal of ^206^Pb was used for peak-centering reference. Each measurement consists of 240 cycles, with a total analytical time of about 20 min. NIST SRM610 glass was used to calibrate the relative yield of different electron multipliers and evaluate the external reproducibility. On the basis of 29 analyses on NIST610 glass under the same analytical conditions, the ^207^Pb/^206^Pb measurements have an SD (1 SD) of 0.78% with ^206^Pb intensity averaged at 114 cps. The possible SIMS instrumental mass fractionation of Pb isotopes around 0.2% (*49*) was propagated to the uncertainty of single-spot ^207^Pb/^206^Pb analysis.

The second session focused on the Pb isotope measurement of silicates (mainly plagioclase and pyroxene) and matrix. A Köhler illumination mode was used to produce a primary beam of about 30 nA O_2_^−^ to a roughly 30-μm size. Before each measurement, an area of 25 μm around the spot location was raster scanned for 120 s to remove the carbon coating and minimize possible surface contamination. The multicollector mode with four electron multipliers was used to measure ^204^Pb^+^ (L2), ^206^Pb^+^ (C), ^207^Pb^+^ (H1), and ^238^U^16^O^+^ (H2). Exit slit 3 was used, with an MRP of 8000 (50% peak height), sufficient to resolve Pb from known molecular interferences. Each measurement consisted of 10 cycles. On the basis of 35 analyses on NIST610 glass under the same analytical conditions, the ^207^Pb/^206^Pb measurements show 1 SD of 0.22% with ^206^Pb intensity averaged at 9230 cps. The number of SIMS Pb isotope analyses for each type of clast used in this study is summarized in table S2.

The background counts for each channel were measured at regular intervals during each session by using deflector and aperture settings that effectively blank both primary and any residual secondary beams. The data were processed using in-house SIMS data reduction spreadsheets and the Excel add-in Isoplot [version 4.15; (*50*)]. In brief, the Pb isotopic compositions measured in each sample are interpreted as representing a mixture between three main components: (i) initial Pb present in the silicate melt when it crystallized; (ii) radiogenic Pb formed by the decay of U in the silicate after crystallization; and (iii) terrestrial contamination. These end-member components define a triangular array of points on a plot of ^207^Pb/^206^Pb versus ^204^Pb/^206^Pb (Fig. 1B).

### Three-stage modeling calculations and Monte Carlo simulations

The first stage of Pb isotopic evolution that began with the formation of the Solar System, was defined by calcium aluminum inclusion formation at 4567 Ma (*t*_0_) (*27*). This time extended to the time of Moon formation (*t*_1_) with μ_0_ = 8. It makes very little contribution to the radiogenic Pb of the lunar mantle and resulting lunar silicate reservoirs. The second stage represents the duration of the LMO, starting from *t*_1_ to the termination of the LMO, *t*_2_, assumed to be the onset of urKREEP reservoir separation. The μ value of the bulk Moon at this stage is poorly constrained (μ_1_). Older lunar formation requires more moderate μ_1_ values (e.g., 462 ± 46 when *t*_1_ = 4500 Ma) (*15*) and younger lunar formation requires higher μ_1_ values [e.g., (*31*)]. The third stage is created by the formation of the urKREEP reservoir and evolves until 4406 Ma (*t*_3_). The μ value of the urKREEP is even less constrained (μ_2_). Previous estimates suggest that the urKREEP reservoir should have μ values in the range of several thousands (*15*, *31*, *32*). Thus, we keep the μ_1_ value to no more than 1000 and μ_2_ value to no less than μ_1_ in our simulations. We show the evolution curves for *t*_1_ = 4500 Ma and *t*_1_ = 4430 Ma when the bulk Moon μ value is 460 (μ_1_ = 460) (Fig. 2A). The μ_2_ value is a function of the Moon formation time (*t*_1_) and the LMO duration (*t*_1_-*t*_2_). (Fig. 2B).

Equations used in three-stage Pb model are

Pb206Pb204=Pb206Pb204CDT+μ0(eλ1t0−eλ1t1)+μ1(eλ1t1−eλ1t2)+μ2(eλ1t2−eλ1t3)

Pb207Pb204=Pb207Pb204CDT+μ0(eλ2t0−eλ2t1)+μ1(eλ2t1−eλ2t2)+μ2(eλ2t2−eλ2t3)137.818

where Pb206Pb204CDT and Pb207Pb204CDT represent the Pb isotopic composition of CDT (*28*), λ_1_ = 1.55125 × 10^−10^, λ_2_ = 9.8485 × 10^−10^, and μ = ^238^U/^204^Pb.

We use a Monte Carlo approach to simulate the corresponding variations in μ_1_, μ_2_, *t*_1_, and *t*_2_ within a three-stage Pb evolution model. The simulations explore the parameter space through uniform random sampling, with μ_1_ ranging between μ_0_ and 1000, μ_2_ > μ_1_. The temporal parameters *t*_1_ and *t*_2_ are selected from the geological time interval spanning from 4500 to 4406 Ma.

To constrain the HIP value, we plotted the probability distribution for ^204^Pb/^206^Pb and ^207^Pb/^206^Pb ratios and examined their relationship with the μ_2_. We found that the HIP value decreases with increasing μ_2_, and approaches to a constant value (^204^Pb/^206^Pb ≥ 0.00578; ^207^Pb/^206^Pb ≥ 1.74670) when μ_2_ is large enough (e.g., 1,100,000) (fig. S6).

## Supplementary Materials

### The PDF file includes:

Figs. S1 to S8 Legends for tables S1 to S3 References
