# Supplementary material for: Lead isotopic evidence for an old and rapid lunar magma ocean
Source: Sci Adv. 2025 Aug 6;11(32):eadu5111. doi: 10.1126/sciadv.adu5111 (PMC12327474; doi:10.1126/sciadv.adu5111)
Supplement: Supplementary file 1 — Figs. S1 to S8 Legends for tables S1 to S3 References [file sciadv.adu5111_sm.pdf]

Science Advances
AAAS

Supplementary Materials for

Lead isotopic evidence for an old and rapid lunar magma ocean

Ya-Wen Zhang *et al.*

Corresponding author: Shui-Jiong Wang, wsj@cugb.edu.cn; Qiu-Li Li, liqiuli@mail.iggcas.ac.cn

Sci. Adv. **11**, eadu5111 (2025)
DOI: 10.1126/sciadv.adu5111

The PDF file includes:

Figs. S1 to S8
Legends for tables S1 to S3
References

Other Supplementary Material for this manuscript includes the following:

Tables S1 to S3

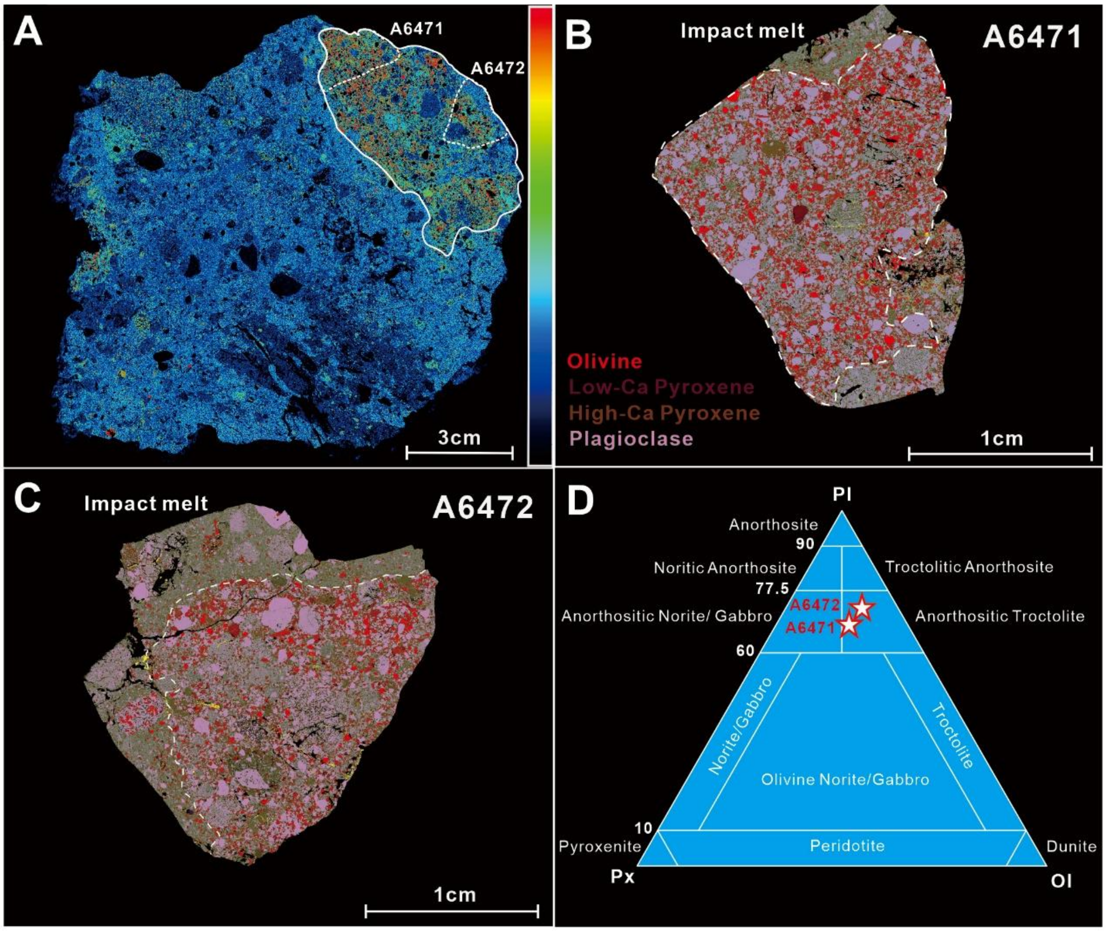

fig. S1 Mineralogy of the troctolitic clast from NWA 14729. (**A**) Magnesium element intensity map of NWA14729 collected with a scanning µXRF mapping analysis. The anorthositic troctolite clast is outlined with white solid line. Rock chips (A6471 and A6472) are outlined with dashed lines. (**B**) and (**C**) are colored elemental distribution maps of rock chips A6471 and A6472. White dashed lines roughly delineate the boundary between the protolith and the impact melts. (**D**) Classification scheme of lunar rocks modified from (*51*). Stars represent compositions of the protoliths in chips A6471 and A6472.

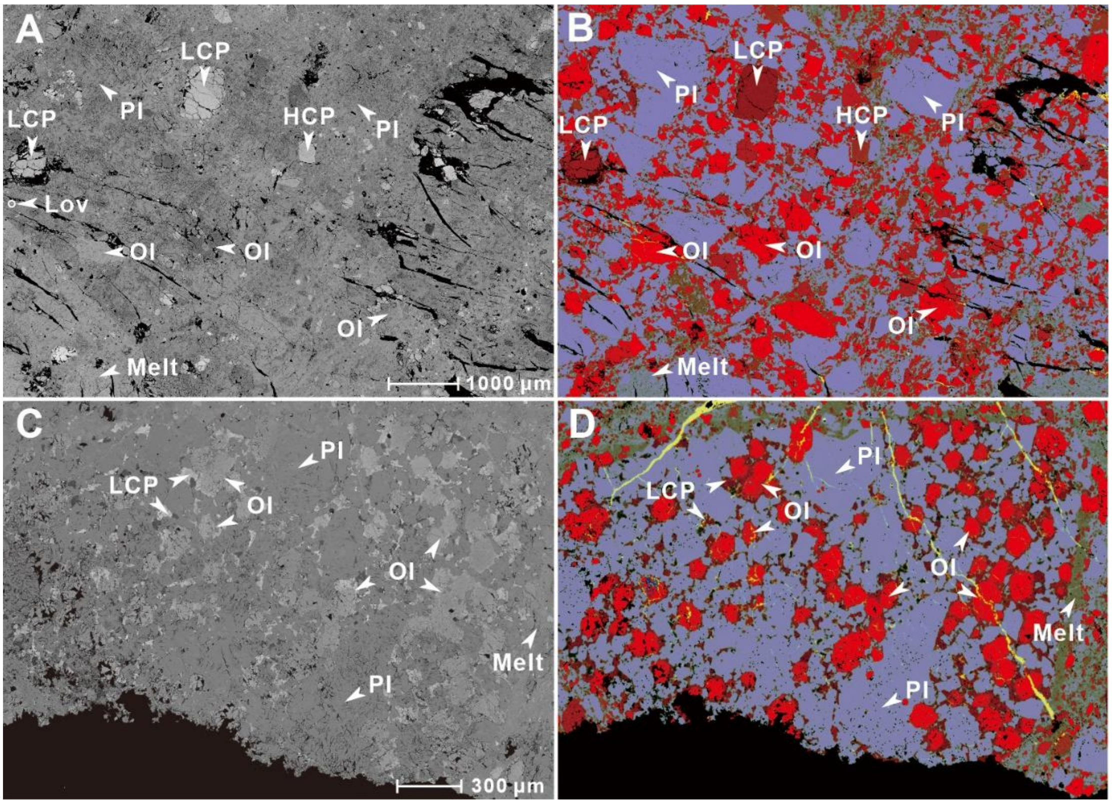

fig. S2 Representative cumulate textures in the troctolitic clast from NWA 14729. (**A**) and (**C**) are backscattered electron images. (**B**) and (**D**) are colored maps revised from TIMA elemental mapping with different phases in individual color. Euhedral plagioclase grains have grain sizes ranging from 0.4 to 2 mm; mafic minerals are dominated by olivine with few low-Ca pyroxene and high-Ca pyroxene, and have generally smaller grain sizes (0.1-0.5 mm). The cumulate olivines are surrounded by a low-Ca pyroxene overgrowth rim suggesting reaction of olivine with silicate melts. Ol, olivine; LCP, low-Ca pyroxene; HCP, high-Ca pyroxene; Pl, plagioclase; Lov, loveringite.

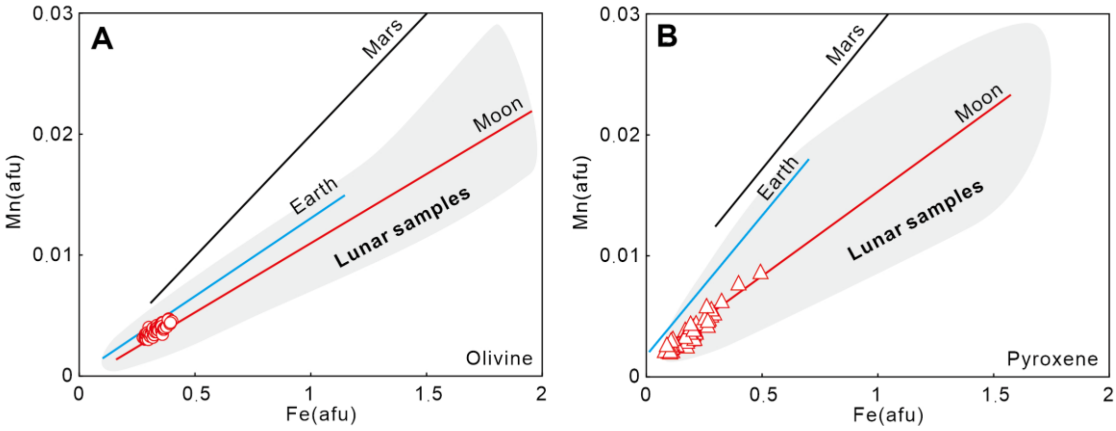

fig. S3 Fe versus Mn (atoms per formula unit) plot of olivine (A) and pyroxene (B) in NWA 14729. The composition fields and trend lines of lunar samples are modified from (*52*). Trends for other planetary bodies are from (*53*).

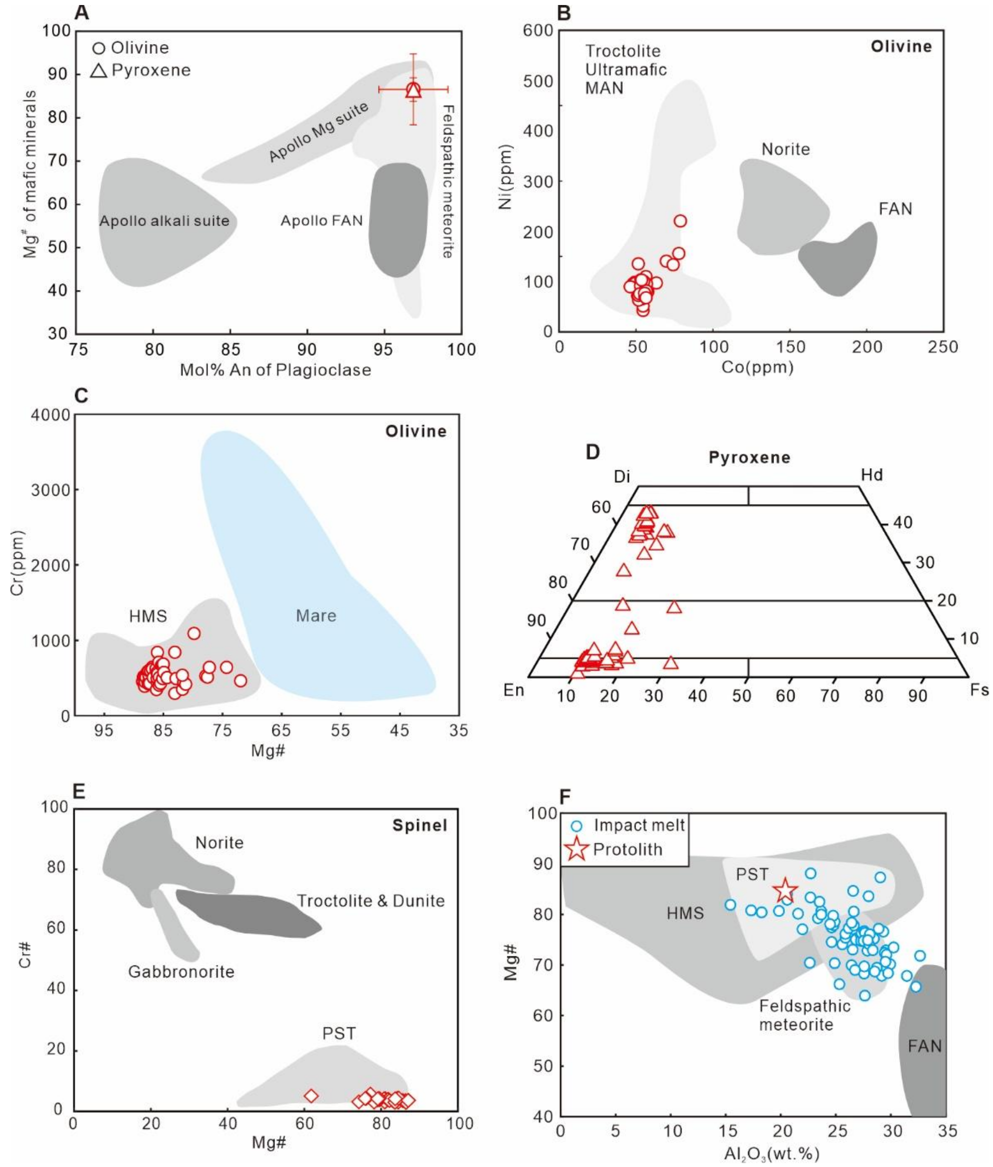

fig. S4 Mineral and glass compositions of the anorthositic troctolite clast. (**A**) Graph of anorthite component (mol%) of plagioclase versus Mg# of mafic minerals. The bars represent the two standard errors. Compositional fields for the Apollo samples and feldspathic meteorites are from (*54*) and (*55*) (after (*56*)) respectively. (**B**) Plot of Ni versus Co in parts per million in olivine compared to olivine from Mg-suite lithologies and ferroan anorthosites (modified after (*25*) and references therein). (**C**) Plot of Cr in parts per million versus Mg# of olivine, compared to olivine in Mg-suite lithologies and mare basalts, after (*57*) and references therein. (**D**) Pyroxenes compositions. (**E**) Plot of Cr# versus Mg# of spinel compared to Mg-suite lithologies after (*58*) and references therein. (**F**) Mg# versus Al_2O_3 contents of impact melts compared to the reconstructed composition of anorthositic troctolite clast in this study, Mg-suite lithologies, ferroan anorthosites, and feldspathic meteorites. The composition of the protolith is calculated based on modal mineral proportions and mineral densities as follow: 4.07 g/cm^3 for olivine, 3.40 g/cm^3 for pyroxenes and 2.09 g/cm^3 for plagioclase. The compositions of Mg-suite lithologies, ferroan anorthosites and feldspathic meteorites are from The Lunar Sample Compendium at https://curator.jsc.nasa.gov/lunar/lsc/, as well as (*59*) and references therein. HMS, Mg-suite lithologies; PST, pink spinel troctolite; FAN, ferroan anorthosite.

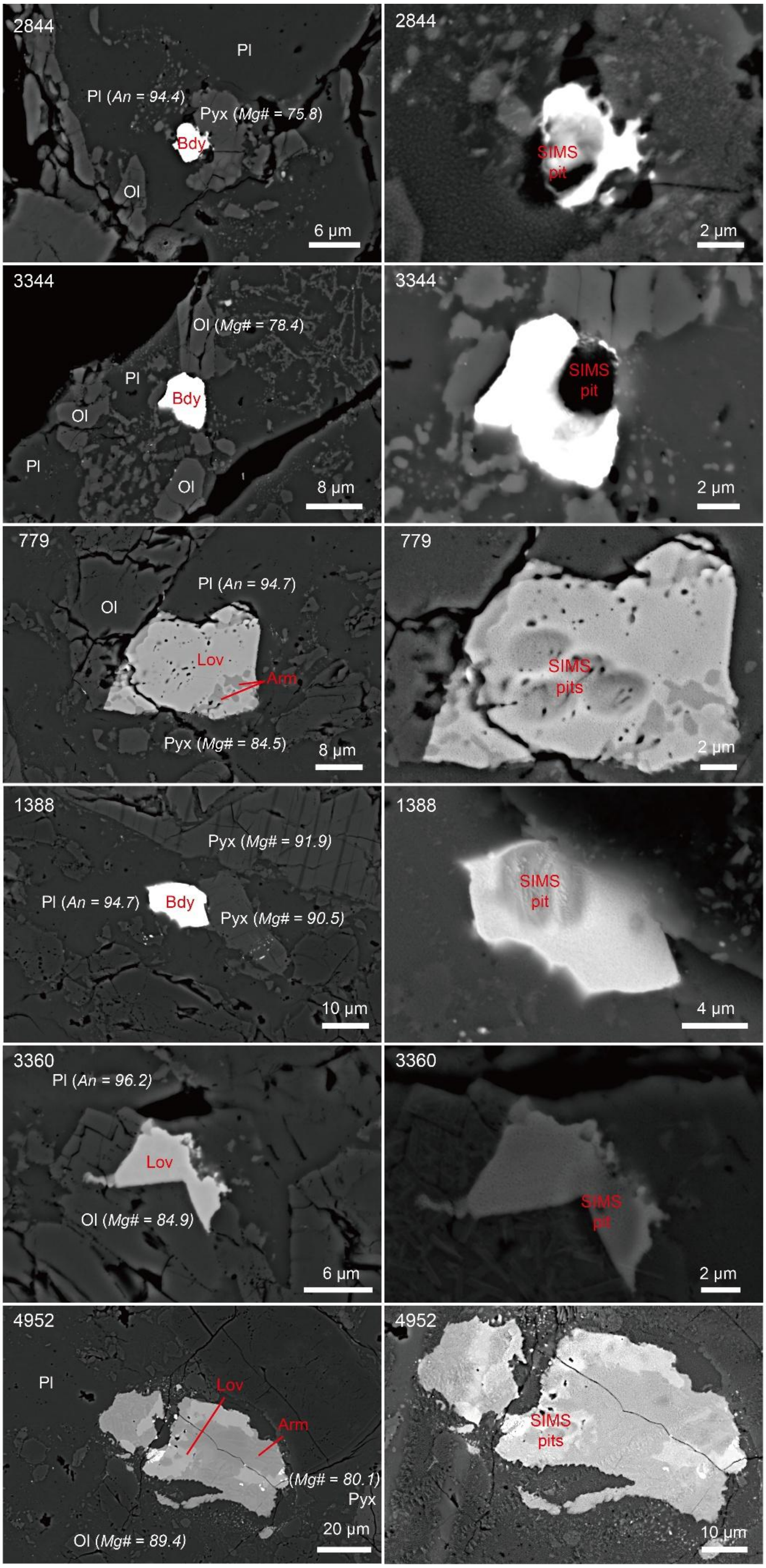

2844
Pl
Pl (An = 94.4)
Pyx (Mg# = 75.8)
Bdy
Ol
6 μm
2844
SIMS pit
2 μm
3344
Ol (Mg# = 78.4)
Pl
Bdy
Ol
Pl
Ol
8 μm
3344
SIMS pit
2 μm
779
Pl (An = 94.7)
Ol
Lov
Arm
Pyx (Mg# = 84.5)
8 μm
779
SIMS pits
2 μm
1388
Pyx (Mg# = 91.9)
Pl (An = 94.7)
Bdy
Pyx (Mg# = 90.5)
10 μm
1388
SIMS pit
4 μm
3360
Pl (An = 96.2)
Lov
Ol (Mg# = 84.9)
6 μm
3360
SIMS pit
2 μm
4952
Pl
Lov
Arm
(Mg# = 80.1)
Pyx
Ol (Mg# = 89.4)
20 μm
4952
SIMS pits
10 μm

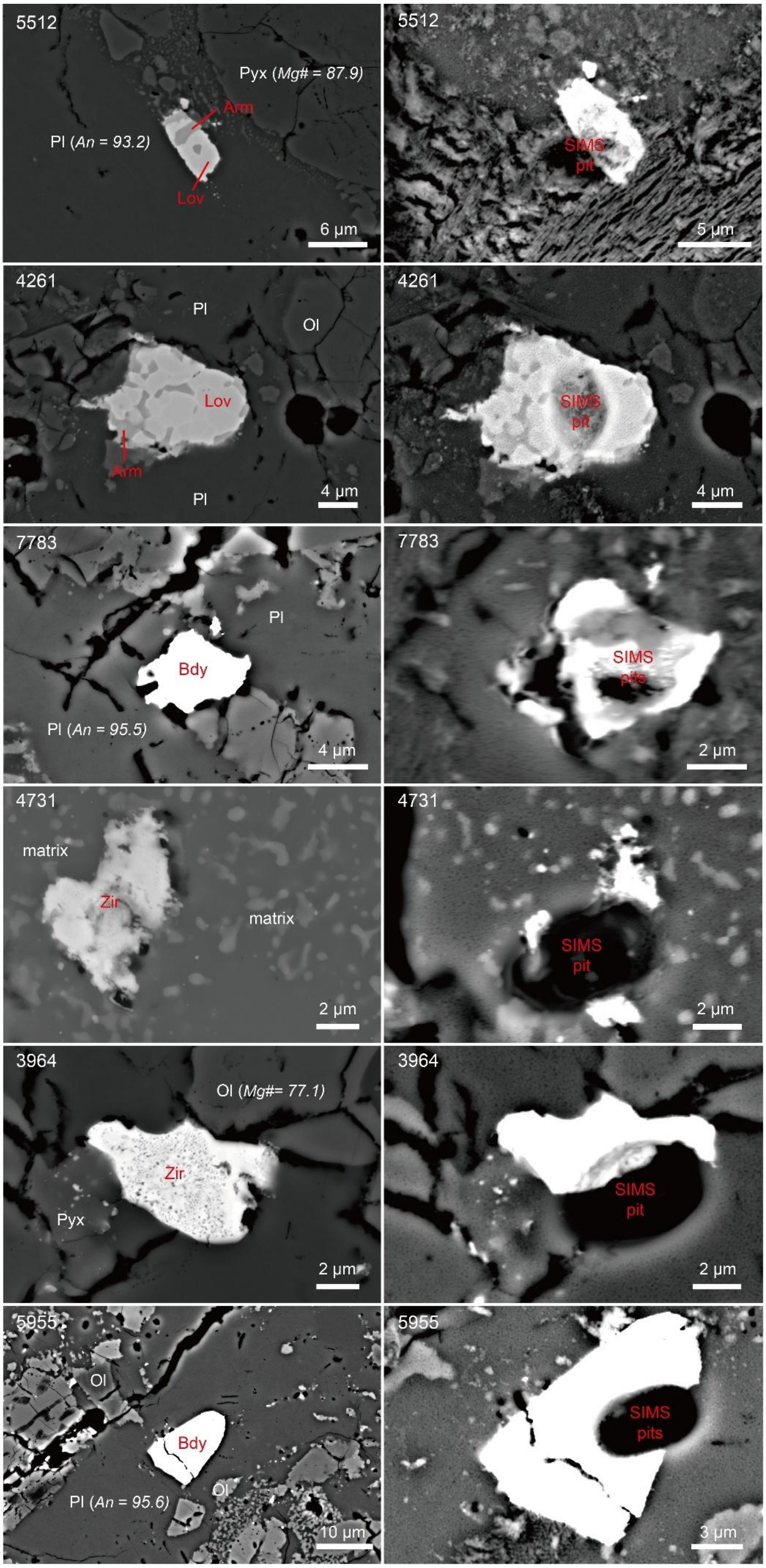
5512
Pyx (Mg# = 87.9)
Arm
Pl (An = 93.2)
Lov
6 μm
5512
SIMS pit
5 μm
4261
Pl
Ol
Lov
Arm
Pl
4 μm
4261
SIMS pit
4 μm
7783
Pl
Bdy
Pl (An = 95.5)
4 μm
7783
SIMS pits
2 μm
4731
matrix
Zir
matrix
2 μm
4731
SIMS pit
2 μm
3964
Ol (Mg#= 77.1)
Zir
Pyx
2 μm
3964
SIMS pit
2 μm
5955
Ol
Bdy
Ol
Pl (An = 95.6)
10 μm
5955
SIMS pits
3 μm

fig. S5 Back-scattered electron (BSE) images of representative dated Zr-phases in NWA 14729. Left, before SIMS analyses; right, after SIMS analyses. Bdy, baddeleyite; Zir, zircon; Lov, Loveringite; Arm, armalcolite; Cpx, clinopyroxene; Pl, plagioclase; Ol, olivine. Numbers in the upper left corner represent the number of the Zr-phases.

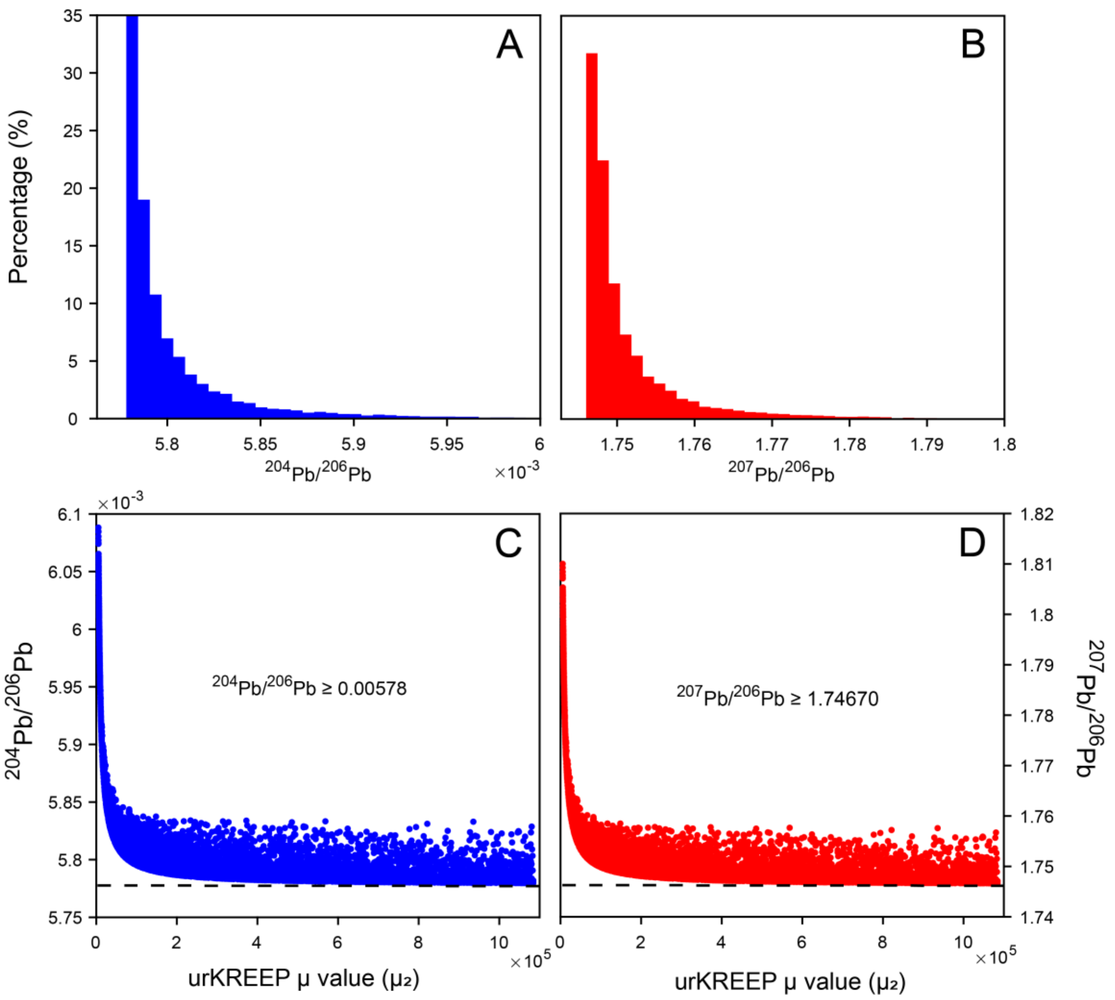

fig. S6 Probability distribution of ^{204}Pb/^{206}Pb and ^{207}Pb/^{206}Pb ratios (A, B) and their correlations with μ_2 (C, D). μ_2 is ca. 1,060,000 when t_1 is ca. 4406 Ma.

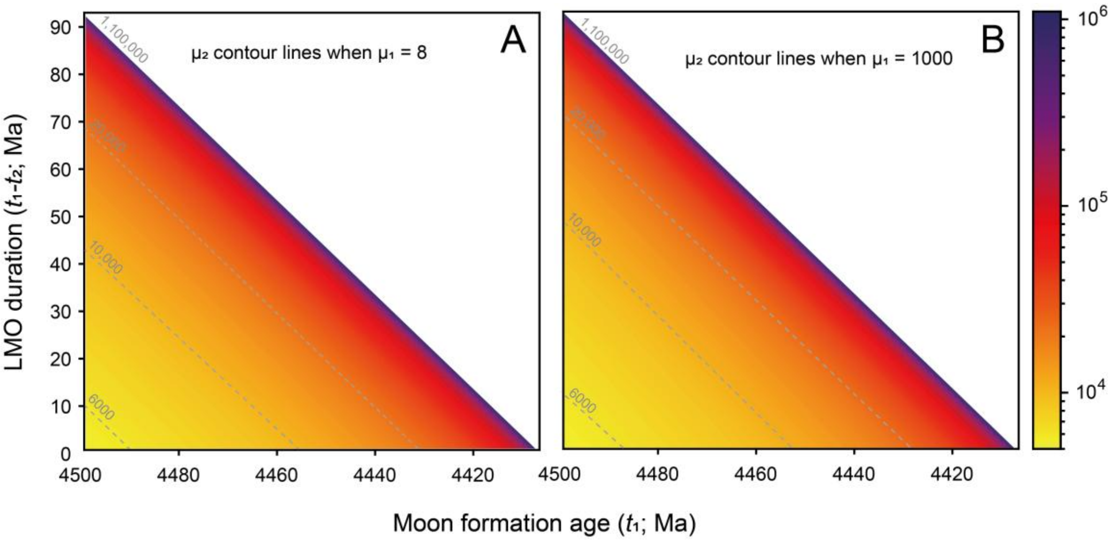

fig. S7 Monte Carlo-derived modeled μ_2 values as a function of the Moon formation time (t_1) and the LMO duration (t_1-t_2). (**A**) and (**B**) are contour lines of μ_2 with different μ_1.

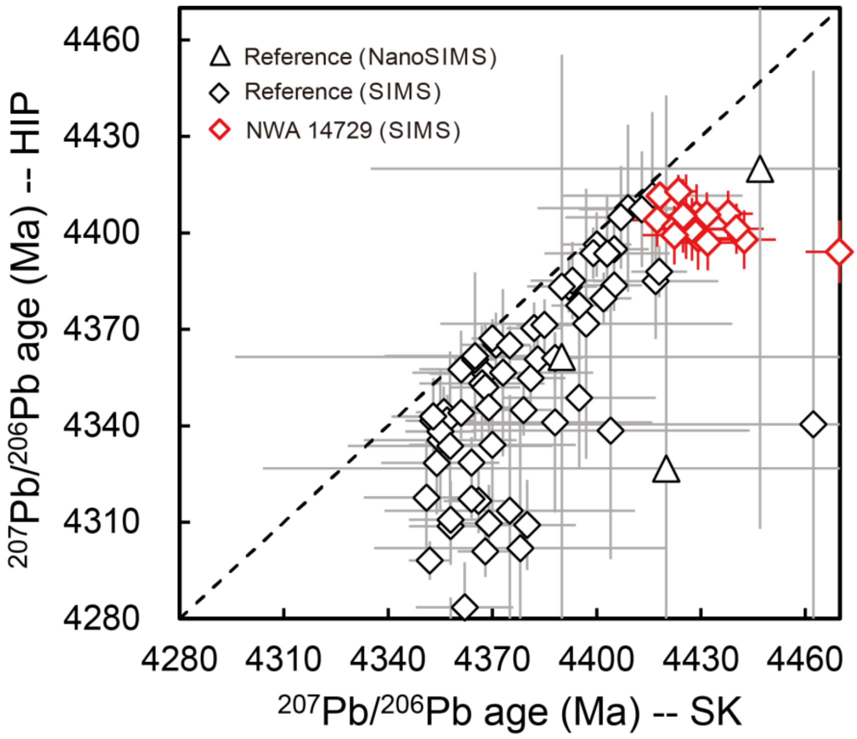

fig. S8 ^{207}Pb/^{206}Pb ages corrected by HIP versus SK for Zr-phases in the reference and this study. ^{207}Pb/^{206}Pb data for Zr-phases older than 4350 Ma were from (*11, 13, 16, 19-22, 41*). Corrections were made using SK (modern terrestrial Pb composition) (*26*) and the high initial Pb (HIP) isotopic composition (^{204}Pb/^{206}Pb = 0.00578 and ^{207}Pb/^{206}Pb = 1.74670). Error bars represent 2σ. Detailed data are reported in table S2 and S3.

table S1. Average major element content of minerals in NWA14729. All analytical major element data including Zr-phases, olivine, pyroxene, plagioclase and spinel.

table S2. Pb isotopic data of Zr-phases and silicates. Including SIMS Pb-Pb data from Zr-phases and silicates. $^{207}Pb/^{206}Pb$ ages of Zr-phases are corrected from different initial Pb compositions. SK, modern terrestrial Pb composition; CDT, Canyon Diablo Troilite Pb isotopic composition; HIP, our high initial Pb isotopic composition.

table S3. Correction of $^{207}Pb/^{206}Pb$ ages older than 4350 Ma in the literature. Including uncorrected and corrected $^{207}Pb/^{206}Pb$ age data in the literature. SK, modern terrestrial Pb composition; HIP, our high initial Pb isotopic composition.

REFERENCES AND NOTES

1. R. M. Canup, Forming a Moon with an Earth-like composition via a giant impact. *Science* **338**, 1052–1055 (2012).

2. P. H. Warren, The magma ocean concept and lunar evolution. *Annu. Rev. Earth Planet. Sci.* **13**, 201–240 (1985).

3. J. A. Wood, J. S. Dickey Jr, U. B. Marvin, B. N. Powell, Lunar anorthosites. *Science* **167**, 602–604 (1970).

4. L. T. Elkins-Tanton, S. Burgess, Q.-Z. Yin, The lunar magma ocean: Reconciling the solidification process with lunar petrology and geochronology. *Earth Planet. Sci. Lett.* **304**, 326–336 (2011).

5. P. H. Warren, J. T. Wasson, The origin of KREEP. *Rev. Geophys.* **17**, 73–88 (1979).

6. M. Barboni, P. Boehnke, B. Keller, I. E. Kohl, B. Schoene, E. D. Young, K. D. McKeegan, Early formation of the Moon 4.51 billion years ago. *Sci. Adv.* **3**, e1602365 (2017).

7. L. E. Borg, R. W. Carlson, The evolving chronology of moon formation. *Annu. Rev. Earth Planet. Sci.* **51**, 25–52 (2023).

8. M. Maurice, N. Tosi, S. Schwinger, D. Breuer, T. Kleine, A long-lived magma ocean on a young Moon. *Sci. Adv.* **6**, eaba8949 (2020).

9. F. Nimmo, T. Kleine, A. Morbidelli, Tidally driven remelting around 4.35 billion years ago indicates the Moon is old. *Nature* **636**, 598–602 (2024).

10. N. Dauphas, Z. J. Zhang, X. Chen, M. Barboni, D. Szymanowski, B. Schoene, I. Leya, K. D. McKeegan, Completion of lunar magma ocean solidification at 4.43 Ga. *Proc. Natl. Acad. Sci. U.S.A.* **122**, e2413802121 (2025).

11. A. Nemchin, N. Timms, R. Pidgeon, T. Geisler, S. Reddy, C. Meyer, Timing of crystallization of the lunar magma ocean constrained by the oldest zircon. *Nat. Geosci.* **2**, 133–136 (2009).

12. W. R. Premo, M. Tatsumoto, K. Misawa, N. Nakamura, N. Kita, Pb-isotopic systematics of lunar highland rocks (>3.9 Ga): Constraints on early lunar evolution. *Int. Geol. Rev.* **41**, 95–128 (1999).

13. M. Grange, R. Pidgeon, A. Nemchin, N. E. Timms, C. Meyer, Interpreting U–Pb data from primary and secondary features in lunar zircon. *Geochim. Cosmochim. Acta* **101**, 112–132 (2013).

14. L. E. Borg, A. M. Gaffney, C. K. Shearer, A review of lunar chronology revealing a preponderance of 4.34–4.37 Ga ages. *Meteorit. Planet. Sci.* **50**, 715–732 (2015).

15. J. F. Snape, A. A. Nemchin, J. J. Bellucci, M. J. Whitehouse, R. Tartèse, J. J. Barnes, M. Anand, I. A. Crawford, K. H. Joy, Lunar basalt chronology, mantle differentiation and implications for determining the age of the Moon. *Earth Planet. Sci. Lett.* **451**, 149–158 (2016).

16. A. Nemchin, R. Pidgeon, M. Whitehouse, J. P. Vaughan, C. Meyer, SIMS U–Pb study of zircon from Apollo 14 and 17 breccias: Implications for the evolution of lunar KREEP. *Geochim. Cosmochim. Acta* **72**, 668–689 (2008).

17. L. E. Borg, J. N. Connelly, M. Boyet, R. W. Carlson, Chronological evidence that the Moon is either young or did not have a global magma ocean. *Nature* **477**, 70–72 (2011).

18. L. E. Borg, A. M. Gaffney, T. S. Kruijer, N. A. Marks, C. K. Sio, J. Wimpenny, Isotopic evidence for a young lunar magma ocean. *Earth Planet. Sci. Lett.* **523**, 115706 (2019).

19. D. J. Taylor, K. D. McKeegan, T. M. Harrison, Lu–Hf zircon evidence for rapid lunar differentiation. *Earth Planet. Sci. Lett.* **279**, 157–164 (2009).

20. B. Zhang, Y. Lin, D. E. Moser, J. Hao, Y. Liu, J. Zhang, I. R. Barker, Q. Li, S. R. Shieh, A. Bouvier, Radiogenic Pb mobilization induced by shock metamorphism of zircons in the Apollo 72255 Civet Cat norite clast. *Geochim. Cosmochim. Acta* **302**, 175–192 (2021).

21. M. Grange, A. Nemchin, N. Timms, R. Pidgeon, C. Meyer, Complex magmatic and impact history prior to 4.1Ga recorded in zircon from Apollo 17 South Massif aphanitic breccia 73235. *Geochim. Cosmochim. Acta* **75**, 2213–2232 (2011).

22. C. Meyer, I. S. Williams, W. Compston, Uranium-lead ages for lunar zircons: Evidence for a prolonged period of granophyre formation from 4.32 to 3.88 Ga. *Meteorit. Planet. Sci.* **31**, 370–387 (1996).

23. M. Barboni, D. Szymanowski, B. Schoene, N. Dauphas, Z. J. Zhang, X. Chen, K. D. McKeegan, High-precision U-Pb zircon dating identifies a major magmatic event on the Moon at 4.338 Ga *Sci. Adv.* **10**, eadn9871 (2024).

24. J. E. Dickinson Jr, P. Hess, Zircon saturation in lunar basalts and granites. *Earth Planet. Sci. Lett.* **57**, 336–344 (1982).

25. C. K. Shearer, J. Papike, Early crustal building processes on the moon: Models for the petrogenesis of the magnesian suite. *Geochim. Cosmochim. Acta* **69**, 3445–3461 (2005).

26. J. S. Stacey, J. D. Kramers, Approximation of terrestrial lead isotope evolution by a two-stage model. *Earth Planet. Sci. Lett.* **26**, 207–221 (1975).

27. J. N. Connelly, M. Bizzarro, A. N. Krot, Å. Nordlund, D. Wielandt, M. A. Ivanova, The absolute chronology and thermal processing of solids in the solar protoplanetary disk. *Science* **338**, 651–655 (2012).

28. C. Göpel, G. Manhès, C. J. Allègre, U-Pb systematics in iron meteorites: Uniformity of primordial lead. *Geochim. Cosmochim. Acta* **49**, 1681–1695 (1985).

29. R. E. Zartman, B. R. Doe, Plumbotectonics—The model. *Tectonophysics* **75**, 135–162 (1981).

30. J. D. Kramers, I. N. Tolstikhin, Two terrestrial lead isotope paradoxes, forward transport modelling, core formation and the history of the continental crust. *Chem. Geol.* **139**, 75–110 (1997).

31. J. Connelly, A. Nemchin, R. E. Merle, J. Snape, M. J. Whitehouse, M. Bizzarro, Calibrating volatile loss from the Moon using the U-Pb system. *Geochim. Cosmochim. Acta* **324**, 1–16 (2022).

32. J. F. Snape, A. A. Nemchin, M. J. Whitehouse, Q.-L. Li, Y. Liu, N. E. Timms, T. Erickson, G. K. Benedix, Post-imbrium Pb–Pb isochron ages for Apollo basaltic impact melt samples 14078 and 68415. *R. Soc. Open Sci.* **11**, 231963 (2024).

33. M. Touboul, T. Kleine, B. Bourdon, H. Palme, R. Wieler, Late formation and prolonged differentiation of the Moon inferred from W isotopes in lunar metals. *Nature* **450**, 1206–1209 (2007).

34. W. Compston, I. S. Williams, C. Meyer, U-Pb geochronology of zircons from lunar breccia 73217 using a sensitive high mass-resolution ion microprobe. *J. Geophys. Res. Solid Earth* **89**, B525–B534 (1984).

35. A. M. Gaffney, L. E. Borg, Y. Asmerom, The origin of geochemical diversity of lunar mantle sources inferred from the combined U–Pb, Rb–Sr, and Sm–Nd isotope systematics of mare basalt 10017. *Geochim. Cosmochim. Acta* **71**, 3656–3671 (2007).

36. J. K. Dhaliwal, J. M. Day, F. Moynier, Volatile element loss during planetary magma ocean phases. *Icarus* **300**, 249–260 (2018).

37. L. T. Elkins-Tanton, Linked magma ocean solidification and atmospheric growth for Earth and Mars. *Earth Planet. Sci. Lett.* **271**, 181–191 (2008).

38. J. F. Snape, N. M. Curran, M. J. Whitehouse, A. A. Nemchin, K. H. Joy, T. Hopkinson, M. Anand, J. J. Bellucci, G. G. Kenny, Ancient volcanism on the Moon: Insights from Pb isotopes in the MIL 13317 and Kalahari 009 lunar meteorites. *Earth Planet. Sci. Lett.* **502**, 84–95 (2018).

39. F. Tera, G. Wasserburg, U-Th-Pb systematics in lunar highland samples from the Luna 20 and Apollo 16 missions. *Earth Planet. Sci. Lett.* **17**, 36–51 (1972).

40. P. Nunes, M. Tatsumoto, Excess lead in "rusty rock" 66095 and implications for an early lunar differentiation. *Science* **182**, 916–920 (1973).

41. D. Trail, M. Barboni, K. D. McKeegan, Evidence for diverse lunar melt compositions and mixing of the pre-3.9 Ga crust from zircon chemistry. *Geochim. Cosmochim. Acta* **284**, 173–195 (2020).

42. M. Ćuk, D. P. Hamilton, S. J. Lock, S. T. Stewart, Tidal evolution of the Moon from a high-obliquity, high-angular-momentum Earth. *Nature* **539**, 402–406 (2016).

43. D. Stöffler, H.-D. Knöll, U. Marvin, C. Simonds, P. Warren, Recommended classification and nomenclature of lunar highland rocks-a committee report. *Proc. Conf. Lunar Highlands Crust.*, 51–70 (1980).

44. J. Gross, A. H. Treiman, Unique spinel-rich lithology in lunar meteorite ALHA 81005: Origin and possible connection to M3 observations of the farside highlands. *J. Geophys. Res.* **116**, E10009 (2011).

45. A.-C. Zhang, R.-L. Pang, N. Sakamoto, H. Yurimoto, The Cr-Zr-Ca armalcolite in lunar rocks is loveringite: Constraints from electron backscatter diffraction measurements. *Am. Mineral.* **105**, 1021–1029 (2020).

46. J.-H. Li, Q.-L. Li, L. Zhao, J.-H. Zhang, X. Tang, L.-X. Gu, Q. Guo, H.-X. Ma, Q. Zhou, Y. Liu, P. Y. Liu, H. Qiu, G. Li, L. Gu, S. Guo, C. L. Li, X. H. Li, F. Y. Wu, Y. X. Pan, Rapid screening of Zr-containing particles from Chang'e-5 lunar soil samples for isotope geochronology: Technical roadmap for future study. *Geosci. Front.* **13**, 101367 (2022).

47. Y. Liu, X.-H. Li, Q.-L. Li, G.-Q. Tang, Breakthrough of 2- to 3-μm scale U–Pb zircon dating using Cameca IMS-1280HR SIMS. *Surf. Interface Anal.* **52**, 214–223 (2020).

48. Q.-L. Li, X.-H. Li, Y. Liu, G.-Q. Tang, J.-H. Yang, W.-G. Zhu, Precise U–Pb and Pb–Pb dating of Phanerozoic baddeleyite by SIMS with oxygen flooding technique. *J. Anal. At. Spectrom* **25**, 1107–1113 (2010).

49. R. A. Stern, S. Bodorkos, S. L. Kamo, A. H. Hickman, F. Corfu, Measurement of SIMS instrumental mass fractionation of Pb isotopes during zircon dating. *Geostand. Geoanal. Res.* **33**, 145–168 (2009).

50. K. Ludwig, Isoplot 4.15: A Geochronological Toolkit for Microsoft Excel (Berkeley Geochronology Center, 2008).

51. M. A. Wieczorek, B. L. Jolliff, A. Khan, M. E. Pritchard, B. P. Weiss, J. G. Williams, L. L. Hood, K. Righter, C. R. Neal, C. K. Shearer, The constitution and structure of the lunar interior. *Rev. Mineral. Geochem.* **60**, 221–364 (2006).

52. K. H. Joy, M. E. Zolensky, K. Nagashima, G. R. Huss, D. K. Ross, D. S. McKay, D. A. Kring, Direct detection of projectile relics from the end of the lunar basin–forming epoch. *Science* **336**, 1426–1429 (2012).

53. J. Papike, J. Karner, C. Shearer, P. Burger, Silicate mineralogy of martian meteorites. *Geochim. Cosmochim. Acta* **73**, 7443–7485 (2009).

54. M. Lindstrom, U. Marvin, D. Mittlefehldt, Apollo 15 Mg-and Fe-norites-A redefinition of the Mg-suite differentiation trend. *Lunar. Planet Sci. Conf.* **19**, 245–254 (1989).

55. S. M. Elardo, D. F. A. Manosalva, Complexity and ambiguity in the relationships between major lunar crustal lithologies and meteoritic clasts inferred from major and trace element modeling. *Geochim. Cosmochim. Acta* **354**, 13–26 (2023).

56. J. Gross, A. H. Treiman, C. N. Mercer, Lunar feldspathic meteorites: Constraints on the geology of the lunar highlands, and the origin of the lunar crust. *Earth Planet. Sci. Lett.* **388**, 318–328 (2014).

57. S. M. Elardo, D. S. Draper, C. K. Shearer Jr, Lunar Magma Ocean crystallization revisited: Bulk composition, early cumulate mineralogy, and the source regions of the highlands Mg-suite. *Geochim. Cosmochim. Acta* **75**, 3024–3045 (2011).

58. S. M. Elardo, F. M. McCubbin, C. K. Shearer Jr, Chromite symplectites in Mg-suite troctolite 76535 as evidence for infiltration metasomatism of a lunar layered intrusion. *Geochim. Cosmochim. Acta* **87**, 154–177 (2012).

59. J. W. Shervais, J. J. McGee, Petrology of the Western Highland Province: Ancient crust formation at the Apollo 14 site. *J. Geophys. Res.* **104**, 5891–5920 (1999).
